# From Lipid Signatures to Cellular Responses: Unraveling the Complexity of Melanoma and Furthering Its Diagnosis and Treatment

**DOI:** 10.3390/medicina60081204

**Published:** 2024-07-25

**Authors:** Elisa Díaz-Grijuela, Agustín Hernández, Claudia Caballero, Roberto Fernandez, Raquel Urtasun, Marina Gulak, Egoitz Astigarraga, Miguel Barajas, Gabriel Barreda-Gómez

**Affiliations:** 1 Betternostics SL, 31110 Noáin, Spain; ediaz@betternostic.com (E.D.-G.); ahernandez@betternostic.com (A.H.); ccaballero@betternostic.com (C.C.);; 2 IMG Pharma Biotech, Research and Development Division, 48170 Zamudio, Spain; r.fernandez@imgpharma.com; 3 Biochemistry Area, Department of Health Science, Universidad Pública de Navarra, 31006 Pamplona, Spain; raquel.urtasun@unavarra.es (R.U.); miguel.barajas@unavarra.es (M.B.); 4 Cruz Roja Hospital, 48013 Bilbao, Spain; marina-gulak@libero.it

**Keywords:** melanoma, comparative oncology, liquid biopsy, mass spectrometry, lipidomics

## Abstract

Recent advancements in mass spectrometry have significantly enhanced our understanding of complex lipid profiles, opening new avenues for oncological diagnostics. This review highlights the importance of lipidomics in the comprehension of certain metabolic pathways and its potential for the detection and characterization of various cancers, in particular melanoma. Through detailed case studies, we demonstrate how lipidomic analysis has led to significant breakthroughs in the identification and understanding of cancer types and its potential for detecting unique biomarkers that are instrumental in its diagnosis. Additionally, this review addresses the technical challenges and future perspectives of these methodologies, including their potential expansion and refinement for clinical applications. The discussion underscores the critical role of lipidomic profiling in advancing cancer diagnostics, proposing a new paradigm in how we approach this devastating disease, with particular emphasis on its application in comparative oncology.

## 1. Introduction

Cancer is one of the major causes of demise in developed countries, only second to cardiovascular disease [1]. The types of malignancies most prevalent vary with country and year. However, breast cancer and melanoma are frequently found among the ones showing the greatest incidence worldwide [2,3,4]. Furthermore, in the USA, it is predicted that they will become the major types by the year 2040 [5]. This roughly coincides with the prevalence of spontaneous malignancies in other mammals, such as cats and dogs. Nevertheless, in the former species, lymphoma is the most common, while in the latter case, mastocytoma, lymphomas, and hemangiosarcoma show very similar numbers to melanoma [6,7,8,9].

Early, accurate diagnosis is paramount to success in any medical intervention, and this is especially true for cancer. In the case of Non-White individuals, delayed diagnosis of melanoma is more common than in Caucasians and is associated with worse survival rates [10]. A similar situation may occur in the case of vulvar melanoma in women [11]. Recently, the use of dermoscopy has significantly enhanced the early detection and diagnosis of melanoma, providing detailed visualization of pigmented skin lesions [12,13]. Additionally, oxidation markers such as malondialdehyde (MDA) have been highlighted as important biomarkers in melanoma patients. Elevated MDA levels, indicative of lipid peroxidation and oxidative stress, have been consistently reported across various studies (e.g., [14,15,16]), underscoring their potential role in melanoma pathogenesis and progression. On the other hand, misdiagnose is also a source for intervention errors. Difficulties arise when trying to differentiate melanoma from certain types of benign nevi on the basis of usual histopathological criteria. For example, some tumors show overlapping histopathologic similarities with some types of nevi, creating difficulties in distinguishing them even for experienced pathologists [17]. Moreover, the choice of the solid biopsy method used, either shave, punch, or incisional, may alter the estimation of the depth of tumor invasion and may change the recommendations for surgical management in up to 18% of the cases [18]. This is further complicated by the fact that the distinction between benign and malignant nevi does not follow the staining profile of any specific immunohistochemical marker, although Ki-67, pHH3 and p16 are proposed and sometimes used in that respect [17]. In addition to histopathology, imaging techniques based on radiology, ultrasound or magnetic resonance, among others, are also widely used, often prior to a biopsy [19]. Radiological detection of mammary cancer, for example, is long known for its convenience in routine checks, although this technique is cumbersome, potentially dangerous, and it may lead to non-attendance at follow-up appointments [20].

## 2. Diagnostic Methods in Cancer and Melanoma

The panoply of diagnostic methods available for the detection of cancer, and melanoma in particular, is fairly large. However, there are several aspects to take into account when judging the utility of a diagnostic technique. The ability to distinguish between malignant and normal tissue or benign lesions is the first thing that comes to mind. Quantifying the accuracy, sensitivity, and specificity of immunohistochemical techniques is difficult. Nevertheless, some studies have shown that pathologists with >10 years of experience, confronted with the identification of putative melanomas, were accurate in 80% of the cases presented to them, while the sensitivity was estimated to be 91%. However, it was also shown that those figures were heavily influenced by training, dropping to only 62% and 56%, respectively, in the case of less trained personnel, although they already had 1–2 years of experience [21]. Imaging procedures also show variable figures, depending on the actual technique. In a typical study, sensitivity and specificity of mammograms visually analyzed by humans were found to be around 73% and 80%, respectively, while ultrasound figures were 100%, 80%, respectively [22]. The tendency towards the introduction of artificial intelligence is strong in this field. However, to the best of our knowledge, sensitivity and specificity figures for machine-guided diagnostic are still just similar to those obtained by trained human radiologists to date [23].

In addition to reliability parameters, other aspects are important to assess the utility of technology, such as ease of acquisition, secondary effects and risks, cost-effectiveness, or patient suffering, among others. This is obvious in the case of difficult-to-access tumors, such as gliomas, but are also important in less evident others, such as breast cancer. As stated above, discomfort associated to mammography tests may induce non-attendance at follow-up appointments [20]. In the case of melanoma, there is room for improvement in this area too. For example, partial biopsies are frequent and may lead to the underestimation of Breslow thickness, while similarity to nevi can make testing based on solid biopsies challenging in individuals with a large number of suspicious lesions [24]. An alternative to this is the use of liquid biopsies. Urine and blood are easy to obtain using non or minimally invasive procedures and, in the case of blood, it is a tissue that is in intimate contact with nearly all others. This last characteristic allows blood plasma to transport a variety of molecules leaked or released from diseased organs or other tissues in response to health imbalances. It is the correlation in the changes in concentrations of these molecules, with respect to the levels found in healthy organism, what makes them useful biomarkers [25].

## 3. Lipids in Cancer

Cancer cells deviate from normal ones in many aspects, and these differences are vital for maintaining a neoplastic phenotype. Among the hallmarks that have been identified as common in all tumors are important variations in the metabolism [26]. Remarkably, lipid metabolism is one of the recipients of these alterations [27]. Concomitantly, it has long been known that the lipid composition of melanoma cells can be quite distinct, making them identifiable from healthy tissue [28].

Being hydrophobic molecules, lipids are not found in significant concentrations in solution in plasma; rather, they are present in colloidal form. In order to form colloids, they need to associate to specific lipid transport systems (LDL, HDL, chylomicrons) or form microvesicles. Variations exist in lipid profiles between the different types of lipoproteins, as a reflection of physiology. Thus, phospholipids are more abundant in HDL than in VLDL, while the reverse is true for triglycerides [29], in accordance with those lipoproteins belonging to two different lipid transport systems [30]. On the other hand, tissue cells, either normal or tumoral, release microvesicles into the extracellular medium and, eventually, they reach the blood plasma. These vesicles are known as exosomes or extracellular vesicles (EVs). In the case of tumor-derived exosomes, these are known to be quite different from other exosomes and are involved in intercellular crosstalk, with multiple consequences. For example, they may induce changes in healthy tissue favoring metastatic colonization [31,32]. Being biomembrane-based secreted vesicular structures, they carry proteins, nucleic acids, and several other molecules that can be used as cancer biomarkers [33]. Naturally, they can also be a source of lipids that can contribute to lipid biomarker signatures in plasma.

Finally, a constant creation of new membranes and cells, together with the increasing need to maintain activated signaling routes that rely on lipid rafts, make tumor cells avid for certain types of lipids, such as cholesterol. The uptake of this lipid through LDL receptor overexpression and increased internalization leads, on the one hand, to a greater flux of LDL along the tumor cell endocytic pathway [34]. On the other hand, it leads to blood hypocholesterolemia, as observed in various types of cancer, including hematopoietic, bowel, lung, prostate and head and neck cancers [35]. This situation is by no means particular for cholesterol; rather, it is common for many types of lipids, with natural variations among them [36]. Accordingly, plasma lipids are considered as a cancer-specific biomarker source with diagnostic value for some time now [37].

## 4. Interplay between Lipid Metabolism and Cellular Respiration in Cancer

The metabolic signature of cancer cells encompasses alterations in glycolysis, mitochondrial respiration, and lipid and amino acid metabolism. Mitochondria play a crucial role in bioenergetic regulation, metabolism, and apoptosis [38,39]. Moreover, they are interconnected with lipid metabolism pathways, suggesting their involvement in metastatic progression and cancer phenotype, such as the carnitine shuttle system, lipoic acid biosynthesis, and sterol modifications (cholesterol) [40]. Specific studies on melanoma have revealed that alterations in lipid metabolism are closely linked to mitochondrial dysfunction. In melanoma cells, dysregulated lipid metabolism contributes to changes in the mitochondrial membrane composition, impacting bioenergetics and promoting survival and proliferation under stress conditions [41,42]. For instance, increased fatty acid oxidation and elevated levels of certain phospholipids have been observed, which correlate with enhanced mitochondrial activity and resistance to apoptosis [43].

**Figure 1:**
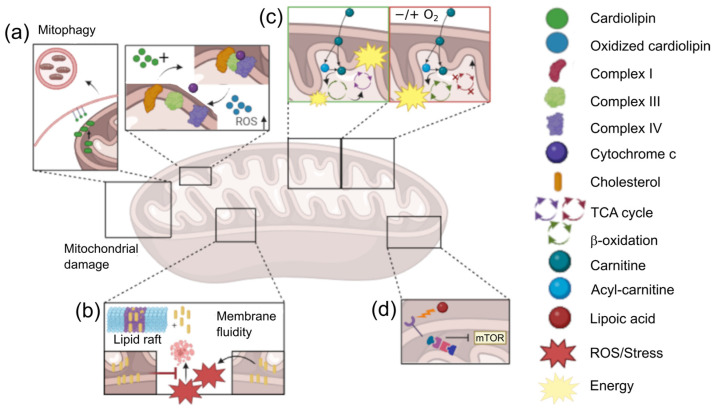
Lipids and mitochondrial metabolism in cancer. (**a**) Cardiolipin involvement in mitochondrial quality control and induction of mitophagy. (**b**) Increased cholesterol levels in tumor mitochondria and the regulation of membrane characteristics. (**c**) Carnitine in cancer; involvement in Warburg effect, Tricarboxylic acid (TCA), and β-oxidation pathways. (**d**) Regulation of mTOR through lipoic acid in cancer.

The lipid composition of mitochondrial membranes (MMs) primarily comprises glycerophospholipids at 75–95%, of which 80% are phosphatidylethanolamines (PEs) and phosphatidylcholines (PCs), and 10–15% are cardiolipins (CLs) [44,45]. CLs account for 20% of the lipid mass in the inner mitochondrial membrane (IMM) [46,47], being essential for the electron transport chain and oxidative phosphorylation (OXPHOS), which is an indispensable process for bioenergetics [48]. Abnormal CL metabolism is associated with various pathologies, including cancer, affecting the structural and functional stability of mitochondria. Abnormal levels of CL interfere with mitochondrial metabolism by altering binding sites in the OXPHOS complexes I, III, and IV, which are necessary for the stability of supercomplexes and the synthesis of acetyl-CoA. Acetyl-CoA is essential for the Krebs cycle, being involved in metastasis and cell migration [47,49]. Additionally, CL participates in mitochondrial quality control through mitophagy. Mitochondrial dysfunction, resulting from increased oxidative stress due to OXPHOS, leads to cellular protection mechanisms, such as apoptosis. In turn, increased OXPHOS leads to lipid oxidation, with the translocation of oxidized CL from the IMM to the outer mitochondrial membrane (OMM), where it is recognized by the mitophagy machinery [45,47,49]. This acts as a survival mechanism, promoting the elimination of damaged mitochondria and preventing apoptosis (Figure 1a).

Cholesterol, which is present in all cellular membranes and is associated with glycosphingolipids in lipid rafts, constitutes approximately 3% of mitochondrial membranes, mainly in the IMM [50,51]. In cancer cells, there is an accumulation of mitochondrial cholesterol, which is correlated with tumor growth and malignancy [52]. This alters the fluidity of MM, affecting transmembrane proteins and increasing the production of reactive oxygen species (ROS) [50,52]. As a result, oxidative phosphorylation is promoted, changing energy metabolism and contributing to chemotherapy resistance by altering mitochondrial apoptosis [51] (Figure 1b).

Carnitine is a key cofactor in metabolism, regulating the acyl-CoA/CoA balance, modulating lipid biosynthesis and degradation, as well as gene expression. It participates in the synthesis of trimethylamine-N-oxide (TMAO), inflammatory processes and fatty acid oxidation (FAO) in the mitochondria [53,54,55]. Carnitine facilitates the transfer of acyl groups across mitochondrial membranes for FAO and ATP production. In cancer, alterations in the expression or activity of carnitine transport are observed, especially in neoplasms with dysregulated fatty acid utilization [54]. Under hypoxia, cancer cells obtain energy from fatty acid oxidation, demonstrating carnitine’s involvement in metabolic plasticity [54]. Furthermore, the rate-limiting enzyme in fatty acid oxidation (FAO) in the metabolic adaptation of cancer is carnitine palmitoyltransferase I (CPTI), which is overexpressed in cancer cells, promoting FAO and the adaptation to metabolic stress [53,54]. This increases NADPH production, facilitating acetyl-CoA generation and providing redox power, which counteracts oxidative stress and promotes cell survival [54,55,56] (Figure 1c).

Lipoic acid (LA) is a mitochondrial cofactor with therapeutic effects, inhibiting tumor proliferation, migration, and invasion, acting as a metal chelator and antioxidant [40,57]. However, studies show that in colon cancer cell lines, LA administration increases OXPHOS, acting as a pro-oxidant and triggering apoptosis by increasing mitochondrial ROS in cancer cells [58,59]. Besides promoting apoptosis, LA modulates mitochondrial metabolism and circulating lipid levels, activating the MAPK signaling pathway and inhibiting mTOR, thereby hindering cell proliferation [40,59] (Figure 1d).

To sum up, lipid metabolism plays a fundamental role in the regulation of mitochondrial function within the context of cancer. Alterations in the lipid composition of mitochondrial membranes, the handling of lipids such as cholesterol and phospholipids, and the modulation of key metabolic pathways such as fatty acid oxidation directly influence mitochondrial bioenergetics and cell signaling. These interactions not only affect the survival and proliferation of cancer cells but also their ability to resist treatments and adapt to adverse environments. 

## 5. Lipid Profile in Melanoma

Lipids play crucial roles in cell biology, functioning as structural components of membranes, signaling molecules, and energy reservoirs. Among the various lipid classes, sphingolipids and glycerolipids are particularly significant in the development and progression of melanoma.

The sphingolipid family is involved in melanoma cell adhesion, metabolic plasticity and aggressiveness [60]. Levels of S-1-P produced by downregulated acid ceramidase induce the loss of E-cadherin in melanoma cells, along with an increase in gangliosides, promoting epithelial–mesenchymal transition (EMT) processes and adhesion to the extracellular matrix, which characterizes the aggressive phenotype of melanoma [60,61,62].

In melanoma cells, the low activity of enzymes responsible for ceramide production has been reported, favoring the consumption of ceramides towards the production of sphingosine-1-phosphate and gangliosides [28,60,63]. Sphingomyelin (SM) synthase activity is notably reduced in melanoma cells compared to melanocytes, resulting in a significant decrease in SM species [64]. This reduction is critical because SM plays a vital role in cell membrane structure and signaling. The decreased levels of SM disrupt membrane integrity and facilitate the production of pro-tumorigenic molecules such as ceramide and sphingosine-1-phosphate, which promote cell proliferation, migration and survival, contributing to melanoma progression and metastasis [65]. Moreover, in stage II patients and proliferative melanoma cells, an increased hydrolysis of ceramide to sphingosine-1-phosphate has been observed [63]. Additionally, a poorer prognosis has been correlated with the downregulation of sphingomyelin synthase 1, which catalyzes the transformation of ceramide to sphingomyelin in patients with metastasis. The lipid profiling of melanocytes, primary melanoma cells, and metastatic melanoma using mass spectrometry has reported a drastic reduction in SM species in melanoma cells compared to melanocytes [66]. 

Phospholipids encompass various lipid classes derived from phosphatidic acid (PA): phosphatidylethanolamine (PE), phosphatidylcholine (PC), phosphatidylinositol and its phosphorylated derivatives (PI, PIP), phosphatidylserine (PS), and phosphatidylglycerol (PG). Abnormalities in glycerophospholipid metabolism, leading to increased levels of PC and PA, favor cell survival by inhibiting apoptosis and inducing EMT and cellular invasion processes. In primary melanoma tumor tissue samples, a significant increase in total PC and PG was revealed compared to nevus melanocytes [67]. Furthermore, mass spectrometry analysis determined that PC, PG, PA, and PI species show increased signals in primary and metastatic melanoma cells [66].

Lipid metabolism is crucial in melanoma due to its role in maintaining cellular membrane integrity, signaling, and energy production [68]. Distinct lipidomic profiles differentiate melanoma from melanocytic nevi, with melanoma cells showing increased levels of specific phospholipids and sphingolipids, which promote tumorigenesis [66]. Lipids such as ceramides and sphingosine-1-phosphate are involved in cell proliferation, migration, and survival, facilitating melanoma expansion and metastasis [69,70]. Understanding these lipid alterations provides insights into melanoma biology and identifies potential therapeutic targets.

## 6. Canine Models in Comparative Melanoma Research

Comparative oncology models, such as canine melanoma, provide significant insights into the role of lipids in tumor biology, helping to elucidate mechanisms that support melanoma development and progression. The lipidomic profiles of canine models of disease show significant parallels with humans [71,72], providing insights into the role of lipids in tumor biology [73]. In the case of melanoma, the similarities between canine and human disease include genetic mutations, histopathological features, and lipidomic profiles [74,75]. Etiologically, both species exhibit mutations in key oncogenes and tumor suppressors such as TP53 and NRAS. Histologically, melanoma in dogs and humans displays similar cellular morphologies and patterns of invasion [75].

The canine models of disease, including spontaneous cancer, offer unique advantages for translational research, leading swiftly to new or improved methods. For instance, the development of hematopoietic cell transplantation for malignant and non-malignant blood diseases in humans and dogs has gained from a parallel development with two-way inputs [76]. Further, other preclinical studies have shown that therapies that are successful in canine models, such as immunotherapies and targeted inhibitors, often translate well to human clinical trials, thereby accelerating the development of effective treatments and improving patient outcomes [77,78]. On the other hand, the study of melanoma in classical murine systems finds difficulties in modelling human tumor heterogeneity and faces important differences in melanocyte locations between species (epidermis in humans and dermis in mice) [75,79]. Additionally, the low efficacy for pharmacological response studies in phase III is well-known [75]. Therefore, better and spontaneous tumor models are of great interest for studying innovative therapies in vivo, their effects on the tumor and the patient, and the interactions between tumor cells and the microenvironment [75,80,81].

In dogs, melanoma accounts for 7% of spontaneous neoplasms [75,82], 14–45% of all oral neoplasms [75], and 9–20% of cutaneous tumors [75]. The most common locations include the mouth (62%), skin (27%), digits (6%), and eyes (1%), among others [83]. Notably, there is a predisposition in males for developing oral (mucosal) melanomas [84]. Despite histological and etiological similarities, canine melanomas also show some differences in humans. The most important may be that the primary risk factor for cutaneous melanomas in humans is UV radiation, with a higher risk in individuals with fair skin, or more specifically, those with very low melanin concentrations [85]. In dogs, the reverse is true, and cutaneous melanoma in dogs increases in breeds with dark skin tones (e.g., Schnauzers, Scottish Terriers, Poodles) [75,85]. Additionally, UV radiation is not a risk factor of the same magnitude as it is in humans, as fur acts as a protective factor [85]. Nevertheless, the canine model may be especially appropriate in the case of mucosal melanoma [86]. Around 30% of melanoma cases in dogs and 50% of mucosal melanomas in humans are amelanotic, making diagnosis challenging due to the lack of melanin [87]. Both exhibit high aggressiveness and metastatic potential, primarily affecting lymph nodes and lungs [75,85,86]. This type of melanoma also has the worst prognosis in dogs, with a median survival of 3 months to 2 years, depending on the stage and treatment choice [75,85,86].

Currently, the prognostic method for oral melanoma in dogs, according to the WHO, is based on tumor size and the presence of distal or regional metastasis. Furthermore, as in humans, prognostic parameters include nuclear atypia, mitotic count, the presence of lymphatic invasion, and the Ki-67 index [86,88,89,90]. Therefore, there are no validated biomarkers capable of providing a reliable prognosis.

The relevance of using domestic animals for comparative oncology studies has increased over the past decade, primarily due to the rise of the One Health movement, but also because of the advantages they present over conventional in vivo models [79,91]. In 2005, the dog genome was sequenced, marking the beginning of comparative studies of various diseases [92,93]. It has been demonstrated that the canine genome has high homology with the human genome. Despite the existence of specific mutations in human melanoma that are absent in canine melanoma, and vice versa, the genomic similarities suggest that the evolutionary lines of dogs and humans are more similar in terms of nucleotide divergence and reorganization than those of humans and rodents [92]. Not surprisingly then, lipidomic analyses also reveal comparable alterations in lipid species, particularly in phospholipids and sphingolipids, underscoring the similarities in the alterations in the metabolic pathways involved in melanoma progression [74]. However, there is clearly room for more studies on canine lipidomics in the different subtypes of melanoma. Given the critical role of lipid metabolism in cancer progression, invasion, and metastasis, this field warrants more extensive investigation.

## 7. Lipidomics and Machine Learning

**Figure 2:**
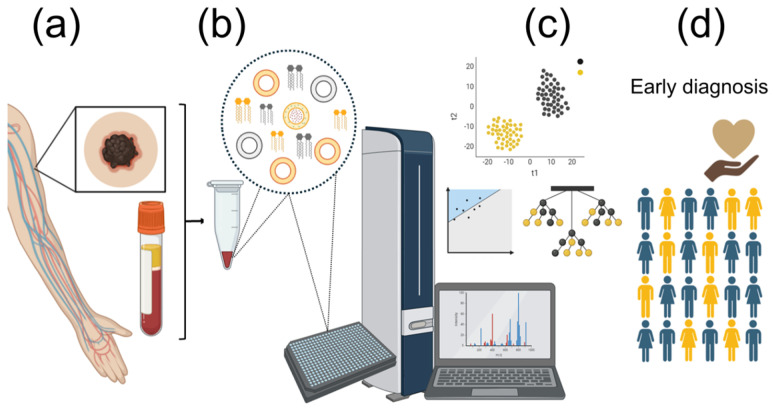
Graphic representation of the process of using mass spectrometry for untargeted lipidomic analysis in the early diagnosis of cancer. (**a**) Sample collection. (**b**) Lipid profile analysis using mass spectrometry. (**c**) Data analysis using machine learning algorithms. (**d**) Early diagnosis of cancer.

In chemical terms, lipids are usually divided in classes, including phosphatidylcholines (PC) or sterol esters. For many purposes, such as understanding the overall biomembrane structure, this level of detail is enough. However, each variation in the molecule resulting from inclusion of a different fatty acid or an ether bond, for example, results in a different species, which is chemically and physiologically different, with some species being more abundant than others. For instance, the most abundant phospholipid species in the plasma membrane tends to be 1-stearoyl-2-oleoyl-sn-phosphatidylcholine (abbreviated PC 18:0/18:1, or often simply, PC 36:1). However, its analogous plasmalogen, 1-O-1′-(Z)-octadecenyl-2-oleoyl-sn-glycero-3-phosphocholine (PC 36:1; O), is less abundant and shows patently different membrane behaviors [94]. Untargeted lipidomics is the individual identification and quantitation of the whole set of lipid species in a sample. This is usually attained using direct mass spectrometry approaches, such as MALDI-TOF or ESI [67,95,96] (Figure 2). Alternatively, lipid-class separation techniques, such as UHPLC, may be associated to MS to improve identification of isobaric species [66,97,98]. 

Needless to say, the vast number of lipid species that can be identified from complex samples, such as blood plasma, require solid, multivariate statistical processing to reach meaningful conclusions. It is out of the scope of this review to describe the plethora of different mathematical approaches employed by lipidomics researchers. Nevertheless, we will try to provide some simple descriptions when possible. The reader is referred to more authoritative works published elsewhere for both multivariate statistics and machine learning algorithms [99,100,101,102]. The reader is also advised that there exists a plethora of method modifications that can increase the difficulties of precisely identifying the actual methods used. 

The present relatively easy use of computer-aided multivariate statistics makes the analysis of large sets of data amenable and, as a consequence, attractive. Not surprisingly, the first analysis of data obtained from lipidomic studies is usually conducted utilizing dimension reduction methods, such as Principal Component Analysis (PCA), Partial Least Squares (PLS), Maximum Margin Criterion (MMC), or their derivatives. This simplifies the analysis of a large number of variables (the levels of individual lipid species) obtained from a single experimental case and permits clustering by graphical means. In many studies, this is enough to show useful differences in lipid composition between healthy and pathological tissues. For example, in a case of hepatocellular carcinoma, the exploratory analysis was performed using PCA, but later, PLS-DA (Partial Least Square Discriminant Analysis, a variant based on PLS) and SIMCA (Soft Independent Modelling of Class Analogy) clustering models were constructed [103]. Similarly, OPLS-DA (Orthogonal PLS-DA) was used to successfully cluster healthy individual plasma from that of patients suffering from pancreatic [104] or papillary thyroid cancer [105]. PLS also helped in the discovery of possible lipid biomarkers in melanoma using cell lines [106] and separated grades in meningioma biopsies [107]. In dogs, OPLS was found superior to PCA in distinguishing mammary tumors [108]. Likewise, OPLS and PCA were used successfully in a more recent study to create models discriminating nevi from primary and metastatic human melanoma [66].

However, it has been the recent development of algorithms capable of autonomously deciding the tests and parameters to be used in mathematical modelling (machine learning, ML) that has set a different ball in motion. A PubMed search using “machine learning”, “diagnosis”, and “cancer” as keywords for papers published since January 2020 offered 8927 results in June 2024, but only 2821 in the previous twenty years (2000–2020). ML algorithms used in lipidomics are nearly exclusively supervised and aim at clustering. Supervised algorithms need the data sets to be divided into training and validating groups. The first group is fed to the chosen ML algorithm to obtain the parameters necessary to construct a second algorithm, a mathematical model able to classify individual cases into different groups on the basis of their associated data. The second data group measures how successful the constructed algorithm is in its predictions when facing an unknown-type case. A list with several lipidomics studies published between 2021 and 2024, using different ML algorithms can be found in Table 1.

ML algorithms may be separated into linear and non-linear types. Linear algorithms assume, at some stage, that the variation relationship between variables is due to a constant factor. Non-linear ones do not make that assumption. Linear algorithms, such as Multiple Linear Regression (MLR) or Logistic Regression (LR) are mathematically simpler. LR is a dichotomic popular algorithm and has been successfully used when classifying gastric lesions using plasma biopsies [117,118] (Table 1). In the case of the 2021 study, the figures for specificity (true negative rate) and sensitivity (true positive rate) obtained were 93.8% and 95.0%, respectively. Moreover, the Receiver Operating Characteristic Area Under the Curve (ROC AUC, or diagnostic accuracy) reached a remarkable 94.4% [117]. Similar figures were observed for several of the models built using LR in the 2022 study too [118]. Other linear approaches have been successfully applied to renal carcinoma [112] and mammary cancer [98]. In this last case, solid biopsies were used to train ML Support Vector Machine (SVM) and LR algorithms, with similar results: values for ROC AUC above 95% were obtained. It must be noted that SVM algorithms can be used under linear and non-linear assumptions, but the actual strategy is seldom stated in the manuscripts. Another case of successful use of SVM comes from a study on meningioma [109]. The authors were able to separate three different types of lesions with a diagnostic accuracy of 87%. Other non-linear algorithms are also widely used. The most common ones include *k*-Nearest Neighbor (KNN) and Naïve Bayes (NB), among others (e.g., [67,95,97,109,114]). In any case, it is difficult to predict which ML algorithm will best cluster the cases available from a study. For this reason, and due to their ease of implementation, it is usual to test several algorithms and opt for the best performing one. In several cases, the data provided similar results using a panel of ML algorithms, such as in small cell lung cancer [110] and an ovarian cancer model [97]. Although non-linear algorithms can be considered more general, they do not necessarily fit better in all cases. For example, in melanoma solid biopsies, the linear ML model constructed using LR was found superior to non-linear models constructed using NB and KNN, with the former achieving a perfect classification of the samples [67].

## 8. From Bench to Market

Translation of academic work into diagnostic tools that can be used in the clinics is an arduous and lengthy process. This is due not only to technical difficulties, such as lack of standardization or lipid fragmentation [119,120], but also to the administrative and legal burden associated with such delicate matters. However, lipidomics clearly shows the potential to differentiate between healthy and tumoral tissue for several cancer types [121]. Accordingly, some early examples of success can be found. For instance, in the field of pancreatic cancer, it has been reported that serum lipidomics could be used in the early detection of this pathology [104], and this served as the basis for a patent claim [122]. More directly relevant to melanoma, UHPLC-lipidomics has already shown its potential to provide biomarkers [66]. Lipidomics was also shown to be useful in histological differentiation between healthy tissue, nevi, and melanoma through imaging mass spectrometry [67,123]. As a consequence, a patent was filed for these methods [124]. It is foreseeable that more and further developments will occur in the near future.

## 9. Perspectives

There are several aspects that present opportunities for cancer lipidomics. Some have been known for some time, such as the necessity for normalization and quality control of lipidomic data to ease interpretation [125]. In this respect, several efforts have been made, and the lipidomics community is close to a consensus [119,126,127,128,129,130]. Related to this, shotgun mass spectrometry techniques are widely used for the identification of pathogens in clinical environments, both as an in-house analytical service and as a contract one [131]. Therefore, if shotgun lipidomic approaches are standardized, there is an opportunity to reach the same level of industry development as other omics, such as genomics or proteomics. As an example of the existing gap, a search in LinkedIn in June 2024 showed 4400 companies using genomics in their description, but only three mentioned lipidomics.

Up to this point, most reports have made use of machine learning algorithms. The inclusion of more advanced artificial intelligence algorithms, such as deep learning, is possibly the next step in this area [132,133,134]. Actually, some attempts have already been made in cancer, albeit not in lipidomics [135,136]. In any case, the present state of the art makes the discrimination of certain types of cancer from healthy individuals easy in a dichotomic way. This may suffice in the case of imaging lipidomics, such as in the already mentioned example in breast cancer [95], since, in these cases, the suspected cancer type is known, and healthy tissue from the same individual may be available for a comparison. Possibly, a closer example to implementation is melanoma, since biopsies from lesions are easy to obtain, but their analysis still suffers from a lack of consensus antigenic markers [17], which can be addressed by lipidomics imaging [67]. However, in more ambitious endeavors, such as making use of liquid biopsies for oncological testing, it will be necessary to implement lipidomic strategies that are able to distinguish between several types of cancer and healthy individuals at the same time. To the best of the authors’ knowledge, no work has dealt with this issue yet, and the question remains on whether lipidomics will be able to distinguish between healthy individuals and oncological patients beyond the current capabilities. Be that as it may, proposals exist for the combined use of multi-omics as a plausible solution to the limitations of single-approach strategies [137]. On the other hand, even if limited to a dichotomic answer, an opportunity is clear for liquid biopsy-based lipidomics as a follow up technique, making post-intervention control tests easier, faster, and less invasive.

Lipidomics can be integrated with other ‘omic’ sciences such as genomics, proteomics, and metabolomics to provide a comprehensive understanding of cancer biology [138,139]. Predictive models that combine data from multiple omic layers have demonstrated enhanced accuracy in characterizing tumor subtypes and predicting patient outcomes. For instance, a multi-omic approach has successfully identified biomarkers for the early diagnosis and metastasis potential by incorporating lipidomic data with metabolic markers and protein expression profiles in the serum from prostate cancer patients [140]. In that study, authors reported a ROC AUC of 89% for a Logistic Regression model using just four biomarkers (two proteins, one metabolite, and one lipid) and two pathological variables (T stage and Gleason score). To date, these studies are just emerging, and it still remains to be applied to melanoma. Nevertheless, no doubt that such integrative models will leverage machine learning algorithms to analyze complex datasets, offering a holistic view of cancer pathogenesis and treatment response.

Finally, a natural tendency is seen to employ the power of lipidomics to gain insight into the mechanistical aspects in cancer. For example, alterations in metabolism related to prostate cancer were identified [141] and the actual influence of PIK3CA mutations on lipid metabolism clarified [142] by lipidomic approaches. Nonetheless, more work and efforts are needed to exploit the full potential of lipidomics in cancer research.

Lipidomics holds significant potential in clinical practice, particularly in the context of translational research. By identifying lipid biomarkers specific to melanoma, lipidomics can aid in early diagnosis, prognosis, and personalized treatment strategies. In the future, lipidomic profiling could become part of routine clinical assessments, enabling non-invasive monitoring of disease progression and response to therapy through liquid biopsies. Moreover, targeting lipid metabolic pathways offers novel therapeutic avenues, making lipidomics a valuable tool in precision oncology.

In conclusion, lipidomics, together with machine learning/artificial intelligence approaches, are a promising tool in cancer research and diagnostics. Breakthroughs are expected to occur in the immediate future.

## Figures and Tables

**Table 1:** Lipidomics studies and machine learning algorithms used.

Reference	Cancer Origin	Analyzed Tissue	Acquisition	ML Algorithms	Notes
[67]	Melanoma	Solid biopsy	MALDI-MS	**LR,** NB, SVM	Lipid imaging
[98]	Mammary	Solid biopsy	UHPLC-MS	LASSO, SVM	SRAA
[95]	Mammary	Solid biopsy	DESI	**KNN**	Lipid imaging
[109]	Meningioma	Solid biopsy	LC-HRMS	DT, KNN, LR, NB, RF, **SVM**	
[97]	Mouse ovarian	Solid biopsy	UHPLC-MS	LR, RF, KNN, SVM, VC	SRAA
[110]	Lung	Serum	LC-MS/MS	LR, RF, SVM	Panel of 8 metabolites, SRAA
[111]	Mammary	Serum	LC-MS	**LR**	Tumor metastatic potential
[96]	Liver	Serum	MALDI-MS	**LDA,** LR, MLP, RF, SVM	
[112]	Renal	Serum	UPLC-MS	**LASSO-SVM**	Coupled ML algorithms
[113]	Pancreas	Serum	MALDI-MS	**SVM**	
[114]	Colorectal	Plasma	LC-MS	KNN, PLS, RF, **SVM**	
[115]	Colorectal	Plasma	LC-MS	**MLR-EM,** BRANN	Tumor Stage Classification
[116]	Glioma	Plasma	HPLC-MS	**SVM**	
[117]	Gastric	Plasma	LC/ESI-MS	**LR**	
[118]	Gastric	Plasma	UHPLC-MS	**LR**	

Value in bold, the algorithm showing best results. Abbreviations: BRANN, Bayesian Regularized Artificial Neural Networks; DT, Decision Tree; KNN, *k*-Nearest Neighbor; LASSO, Least Absolute Shrinkage and Selection Operator; LDA, Linear Discriminant Analysis; LR, Logistic Regression; ML, machine learning; MLP, Multilayer Perceptron; MLR-EM, Multiple Linear Regression with Expectation Maximization; NB, Naïve Bayes; RF, Random Forest; SRAA, similar results for all algorithms; SVM, Support Vector Machine; VC, Voting Classifier.

## References

[1] Curtin S.C., Tejada-Vera B., Bastian B.A. (2024). Deaths: Leading Causes for 2021. Natl. Vital Stat. Rep..

[2] Zheng R., Zeng H., Zhang S., Chen T., Chen W. (2016). National Estimates of Cancer Prevalence in China, 2011. Cancer Lett..

[3] Yao C., Billette J.M. (2022). Short-Term Cancer Prevalence in Canada, 2018. Health Rep..

[4] De Angelis R., Demuru E., Baili P., Troussard X., Katalinic A., Chirlaque Lopez M.D., Innos K., Santaquilani M., Blum M., Ventura L. (2024). Complete Cancer Prevalence in Europe in 2020 by Disease Duration and Country (EUROCARE-6): A Population-Based Study. Lancet Oncol..

[5] Rahib L., Wehner M.R., Matrisian L.M., Nead K.T. (2021). Estimated Projection of US Cancer Incidence and Death to 2040. JAMA Netw. Open.

[6] Sleeckx N., de Rooster H., Veldhuis Kroeze E.J.B., van Ginneken C., van Brantegem L. (2011). Canine Mammary Tumours, an Overview. Reprod. Domest. Anim..

[7] Misdorp W., Misdorp1 W. (1996). Veterinary Cancer Epidemiology. Vet. Q..

[8] Blackwood L. (2013). Cats with Cancer: Where to Start. J. Feline Med. Surg..

[9] Grüntzig K., Graf R., Hässig M., Welle M., Meier D., Lott G., Erni D., Schenker N.S., Guscetti F., Boo G. (2015). The Swiss Canine Cancer Registry: A Retrospective Study on the Occurrence of Tumours in Dogs in Switzerland from 1955 to 2008. J. Comp. Pathol..

[10] Brunsgaard E.K., Wu Y.P., Grossman D. (2023). Melanoma in Skin of Color: Part I. Epidemiology and Clinical Presentation. J. Am. Acad. Dermatol..

[11] Wohlmuth C., Wohlmuth-Wieser I. (2021). Vulvar Melanoma: Molecular Characteristics, Diagnosis, Surgical Management, and Medical Treatment. Am. J. Clin. Dermatol..

[12] Marghoob N.G., Liopyris K., Jaimes N. (2019). Dermoscopy: A Review of the Structures That Facilitate Melanoma Detection. J. Am. Osteopath. Assoc..

[13] Argenziano G., Albertini G., Castagnetti F., De Pace B., Di Lernia V., Longo C., Pellacani G., Piana S., Ricci C., Zalaudek I. (2012). Early Diagnosis of Melanoma: What Is the Impact of Dermoscopy?. Dermatol. Ther..

[14] Bisevac J.P., Djukic M., Stanojevic I., Stevanovic I., Mijuskovic Z., Djuric A., Gobeljic B., Banovic T., Vojvodic D. (2018). Association Between Oxidative Stress and Melanoma Progression. J. Med. Biochem..

[15] Sander C.S., Hamm F., Elsner P., Thiele J.J. (2003). Oxidative Stress in Malignant Melanoma and Non-Melanoma Skin Cancer. Br. J. Dermatol..

[16] Karampinis E., Nechalioti P.M., Georgopoulou K.E., Goniotakis G., Roussaki Schulze A.V., Zafiriou E., Kouretas D. (2023). Systemic Oxidative Stress Parameters in Skin Cancer Patients and Patients with Benign Lesions. Stresses.

[17] Abbas O., Miller D.D., Bhawan J. (2014). Cutaneous Malignant Melanoma: Update on Diagnostic and Prognostic Biomarkers. Am. J. Dermatopathol..

[18] Pavri S.N., Clune J., Ariyan S., Narayan D. (2016). Malignant Melanoma: Beyond the Basics. Plast. Reconstr. Surg..

[19] Goyal L., Hingmire S., Parikh P.M. (2006). Newer Diagnostic Methods in Oncology. Med. J. Armed Forces India.

[20] Whelehan P., Evans A., Wells M., MacGillivray S. (2013). The Effect of Mammography Pain on Repeat Participation in Breast Cancer Screening: A Systematic Review. Breast.

[21] Morton C.A., Mackie R.M. (1998). Clinical Accuracy of the Diagnosis of Cutaneous Malignant Melanoma. Br. J. Dermatol..

[22] Badu-Peprah A., Adu-Sarkodie Y. (2018). Accuracy of Clinical Diagnosis, Mammography and Ultrasonography in Preoperative Assessment of Breast Cancer. Ghana. Med. J..

[23] Schaffter T., Buist D.S.M., Lee C.I., Nikulin Y., Ribli D., Guan Y., Lotter W., Jie Z., Du H., Wang S. (2020). Evaluation of Combined Artificial Intelligence and Radiologist Assessment to Interpret Screening Mammograms. JAMA Netw. Open.

[24] Marghoob A.A., Changchien L., DeFazio J., Dessio W.C., Malvehy J., Zalaudek I., Halpern A.C., Scope A. (2009). The Most Common Challenges in Melanoma Diagnosis and How to Avoid Them. Australas. J. Dermatol..

[25] Poulet G., Massias J., Taly V. (2019). Liquid Biopsy: General Concepts. Acta Cytol..

[26] Hanahan D., Weinberg R.A. (2011). Hallmarks of Cancer: The next Generation. Cell.

[27] Schiliro C., Firestein B.L. (2021). Mechanisms of Metabolic Reprogramming in Cancer Cells Supporting Enhanced Growth and Proliferation. Cells.

[28] Portoukalian J., Zwingelstein G., Doré J.-F. (1979). Lipid Composition of Human Malignant Melanoma Tumors at Various Levels of Malignant Growth. Eur. J. Biochem..

[29] Halpern Z., Rubin M., Harach G., Grotto I., Mosor A., Dvir A., Lichtenberg D., Gilat T. (1993). Bile and Plasma Lipid Composition in Non-Obese Normolipidemic Subjects with and without Cholesterol Gallstones. Liver.

[30] Feingold K.R. (2022). Lipid and Lipoprotein Metabolism. Endocrinol. Metab. Clin. N. Am..

[31] Zhang Y., Wang X.F. (2015). A Niche Role for Cancer Exosomes in Metastasis. Nat. Cell Biol..

[32] Costa-Silva B., Aiello N.M., Ocean A.J., Singh S., Zhang H., Thakur B.K., Becker A., Hoshino A., Mark M.T., Molina H. (2015). Pancreatic Cancer Exosomes Initiate Pre-Metastatic Niche Formation in the Liver. Nat. Cell Biol..

[33] Yu W., Hurley J., Roberts D., Chakrabortty S.K., Enderle D., Noerholm M., Breakefield X.O., Skog J.K. (2021). Exosome-Based Liquid Biopsies in Cancer: Opportunities and Challenges. Ann. Oncol..

[34] Nguyen M.K.L., Jose J., Wahba M., Bernaus-Esqué M., Hoy A.J., Enrich C., Rentero C., Grewal T. (2022). Linking Late Endosomal Cholesterol with Cancer Progression and Anticancer Drug Resistance. Int. J. Mol. Sci..

[35] Munir R., Usman H., Hasnain S., Smans K., Kalbacher H., Zaidi N. (2014). Atypical Plasma Lipid Profile in Cancer Patients: Cause or Consequence?. Biochimie.

[36] Irshad R., Tabassum S., Husain M. (2023). Aberrant Lipid Metabolism in Cancer: Current Status and Emerging Therapeutic Perspectives. Curr. Top. Med. Chem..

[37] Yan F., Zhao H., Zeng Y. (2018). Lipidomics: A Promising Cancer Biomarker. Clin. Transl. Med..

[38] Di Gregorio J., Petricca S., Iorio R., Toniato E., Flati V. (2022). Mitochondrial and Metabolic Alterations in Cancer Cells. Eur. J. Cell Biol..

[39] Caino M.C., Altieri D.C. (2016). Molecular Pathways: Mitochondrial Reprogramming in Tumor Progression and Therapy. Clin. Cancer Res..

[40] Srivastava A., Srivastava P., Mathur S., Abbas S., Rai N., Tiwari S., Tiwari M., Sharma L. (2022). Lipid Metabolism and Mitochondria: Cross Talk in Cancer. Curr. Drug Targets.

[41] Falletta P., Goding C.R., Vivas-García Y. (2022). Connecting Metabolic Rewiring With Phenotype Switching in Melanoma. Front. Cell Dev. Biol..

[42] Harel M., Ortenberg R., Varanasi S.K., Mangalhara K.C., Mardamshina M., Markovits E., Baruch E.N., Tripple V., Arama-Chayoth M., Greenberg E. (2019). Proteomics of Melanoma Response to Immunotherapy Reveals Mitochondrial Dependence. Cell.

[43] Zecchin K.G., Alberici L.C., Riccio M.F., Eberlin M.N., Vercesi A.E., Graner E., Catharino R.R. (2011). Visualizing Inhibition of Fatty Acid Synthase through Mass Spectrometric Analysis of Mitochondria from Melanoma Cells. Rapid Commun. Mass. Spectrom..

[44] Horvath S.E., Daum G. (2013). Lipids of Mitochondria. Prog. Lipid Res..

[45] Poulaki A., Giannouli S. (2022). Mitochondrial Lipids: From Membrane Organization to Apoptotic Facilitation. Int. J. Mol. Sci..

[46] Paradies G., Paradies V., Ruggiero F.M., Petrosillo G. (2019). Role of Cardiolipin in Mitochondrial Function and Dynamics in Health and Disease: Molecular and Pharmacological Aspects. Cells.

[47] Ahmadpour S.T., Mahéo K., Servais S., Brisson L., Dumas J.F. (2020). Cardiolipin, the Mitochondrial Signature Lipid: Implication in Cancer. Int. J. Mol. Sci..

[48] Paradies G., Paradies V., De Benedictis V., Ruggiero F.M., Petrosillo G. (2014). Functional Role of Cardiolipin in Mitochondrial Bioenergetics. Biochim. Biophys. Acta.

[49] Praharaj P.P., Naik P.P., Panigrahi D.P., Bhol C.S., Mahapatra K.K., Patra S., Sethi G., Bhutia S.K. (2019). Intricate Role of Mitochondrial Lipid in Mitophagy and Mitochondrial Apoptosis: Its Implication in Cancer Therapeutics. Cell Mol. Life Sci..

[50] Goicoechea L., Conde de la Rosa L., Torres S., García-Ruiz C., Fernández-Checa J.C. (2023). Mitochondrial Cholesterol: Metabolism and Impact on Redox Biology and Disease. Redox Biol..

[51] Ribas V., García-Ruiz C., Fernández-Checa J.C. (2016). Mitochondria, Cholesterol and Cancer Cell Metabolism. Clin. Transl. Med..

[52] Garcia-Ruiz C., Conde de la Rosa L., Ribas V., Fernandez-Checa J.C. (2021). MITOCHONDRIAL CHOLESTEROL AND CANCER. Semin. Cancer Biol..

[53] Qu Q., Zeng F., Liu X., Wang Q.J., Deng F. (2016). Fatty Acid Oxidation and Carnitine Palmitoyltransferase I: Emerging Therapeutic Targets in Cancer. Cell Death Dis..

[54] Melone M.A.B., Valentino A., Margarucci S., Galderisi U., Giordano A., Peluso G. (2018). The Carnitine System and Cancer Metabolic Plasticity. Cell Death Dis..

[55] Console L., Scalise M., Mazza T., Pochini L., Galluccio M., Giangregorio N., Tonazzi A., Indiveri C. (2020). Carnitine Traffic in Cells. Link With Cancer. Front. Cell Dev. Biol..

[56] Farahzadi R., Hejazi M.S., Molavi O., Pishgahzadeh E., Montazersaheb S., Jafari S. (2023). Clinical Significance of Carnitine in the Treatment of Cancer: From Traffic to the Regulation. Oxid. Med. Cell Longev..

[57] Dörsam B., Fahrer J. (2016). The Disulfide Compound α-Lipoic Acid and Its Derivatives: A Novel Class of Anticancer Agents Targeting Mitochondria. Cancer Lett..

[58] Wenzel U., Nickel A., Daniel H. (2005). Alpha-Lipoic Acid Induces Apoptosis in Human Colon Cancer Cells by Increasing Mitochondrial Respiration with a Concomitant O2-*-Generation. Apoptosis.

[59] Bosso M., Haddad D., Al Madhoun A., Al-Mulla F. (2024). Targeting the Metabolic Paradigms in Cancer and Diabetes. Biomedicines.

[60] Carrié L., Virazels M., Dufau C., Montfort A., Levade T., Ségui B., Andrieu-Abadie N. (2020). New Insights into the Role of Sphingolipid Metabolism in Melanoma. Cells.

[61] Yesmin F., Bhuiyan R.H., Ohmi Y., Yamamoto S., Kaneko K., Ohkawa Y., Zhang P., Hamamura K., Cheung N.K.V., Kotani N. (2021). Ganglioside GD2 Enhances the Malignant Phenotypes of Melanoma Cells by Cooperating with Integrins. Int. J. Mol. Sci..

[62] Noujarède J., Carrié L., Garcia V., Grimont M., Eberhardt A., Mucher E., Genais M., Schreuder A., Carpentier S., Ségui B. (2023). Sphingolipid Paracrine Signaling Impairs Keratinocyte Adhesion to Promote Melanoma Invasion. Cell Rep..

[63] Realini N., Palese F., Pizzirani D., Pontis S., Basit A., Bach A., Ganesan A., Piomelli D. (2016). Acid Ceramidase in Melanoma: EXPRESSION, LOCALIZATION, AND EFFECTS OF PHARMACOLOGICAL INHIBITION. J. Biol. Chem..

[64] Bilal F., Montfort A., Gilhodes J., Garcia V., Riond J., Carpentier S., Filleron T., Colacios C., Levade T., Daher A. (2019). Sphingomyelin Synthase 1 (SMS1) Downregulation Is Associated With Sphingolipid Reprogramming and a Worse Prognosis in Melanoma. Front. Pharmacol..

[65] Bataller M., Sánchez-García A., Garcia-Mayea Y., Mir C., Rodriguez I., LLeonart M.E. (2021). The Role of Sphingolipids Metabolism in Cancer Drug Resistance. Front. Oncol..

[66] Perez-Valle A., Abad-García B., Fresnedo O., Barreda-Gómez G., Aspichueta P., Asumendi A., Astigarraga E., Fernández J.A., Boyano M.D., Ochoa B. (2021). A UHPLC-Mass Spectrometry View of Human Melanocytic Cells Uncovers Potential Lipid Biomarkers of Melanoma. Int. J. Mol. Sci..

[67] Huergo-Baños C., Velasco V., Garate J., Fernández R., Martín-Allende J., Zabalza I., Artola J.L., Martí R.M., Asumendi A., Astigarraga E. (2024). Lipid Fingerprint-Based Histology Accurately Classifies Nevus, Primary Melanoma, and Metastatic Melanoma Samples. Int. J. Cancer.

[68] Huang C., Radi R.H., Arbiser J.L. (2021). Mitochondrial Metabolism in Melanoma. Cells.

[69] Herzinger T., Kleuser B., Schäfer-Korting M., Korting H.C. (2007). Sphingosine-1-Phosphate Signaling and the Skin. Am. J. Clin. Dermatol..

[70] Lai M., La Rocca V., Amato R., Freer G., Pistello M. (2019). Sphingolipid/Ceramide Pathways and Autophagy in the Onset and Progression of Melanoma: Novel Therapeutic Targets and Opportunities. Int. J. Mol. Sci..

[71] Kosinska M.K., Mastbergen S.C., Liebisch G., Wilhelm J., Dettmeyer R.B., Ishaque B., Rickert M., Schmitz G., Lafeber F.P., Steinmeyer J. (2016). Comparative Lipidomic Analysis of Synovial Fluid in Human and Canine Osteoarthritis. Osteoarthr. Cartil..

[72] Sieber-Ruckstuhl N.S., Tham W.K., Baumgartner F., Selva J.J., Wenk M.R., Burla B., Boretti F.S. (2022). Serum Lipidome Signatures of Dogs with Different Endocrinopathies Associated with Hyperlipidemia. Metabolites.

[73] Mangraviti D., Abbate J.M., Iaria C., Rigano F., Mondello L., Quartuccio M., Marino F. (2022). Rapid Evaporative Ionization Mass Spectrometry-Based Lipidomics for Identification of Canine Mammary Pathology. Int. J. Mol. Sci..

[74] Manni M.M., Sot J., Arretxe E., Gil-Redondo R., Falcón-Pérez J.M., Balgoma D., Alonso C., Goñi F.M., Alonso A. (2018). The Fatty Acids of Sphingomyelins and Ceramides in Mammalian Tissues and Cultured Cells: Biophysical and Physiological Implications. Chem. Phys. Lipids.

[75] Prouteau A., André C. (2019). Canine Melanomas as Models for Human Melanomas: Clinical, Histological, and Genetic Comparison. Genes.

[76] Graves S.S., Storb R. (2020). Developments and Translational Relevance for the Canine Haematopoietic Cell Transplantation Preclinical Model. Vet. Comp. Oncol..

[77] Dow S. (2020). A Role for Dogs in Advancing Cancer Immunotherapy Research. Front. Immunol..

[78] Oh W., Jung Kim A.M., Dhawan D., Kirkham P.M., Ostafe R., Franco J., Aryal U.K., Carnahan R.H., Patsekin V., Robinson J.P. (2023). Development of an Anti-Canine PD-L1 Antibody and Caninized PD-L1 Mouse Model as Translational Research Tools for the Study of Immunotherapy in Humans. Cancer Res. Commun..

[79] Schiffman J.D., Breen M. (2015). Comparative Oncology: What Dogs and Other Species Can Teach Us about Humans with Cancer. Philos. Trans. R. Soc. Lond. B Biol. Sci..

[80] Abdelmegeed S.M., Mohammed S. (2018). Canine Mammary Tumors as a Model for Human Disease. Oncol. Lett..

[81] Pinho S.S., Carvalho S., Cabral J., Reis C.A., Gärtner F. (2012). Canine Tumors: A Spontaneous Animal Model of Human Carcinogenesis. Transl. Res..

[82] Stevenson V.B., Klahn S., LeRoith T., Huckle W.R. (2023). Canine Melanoma: A Review of Diagnostics and Comparative Mechanisms of Disease and Immunotolerance in the Era of the Immunotherapies. Front. Vet. Sci..

[83] Gillard M., Cadieu E., De Brito C., Abadie J., Vergier B., Devauchelle P., Degorce F., Dréano S., Primot A., Dorso L. (2014). Naturally Occurring Melanomas in Dogs as Models for Non-UV Pathways of Human Melanomas. Pigment. Cell Melanoma Res..

[84] Gardner H.L., Fenger J.M., London C.A. (2016). Dogs as a Model for Cancer. Annu. Rev. Anim. Biosci..

[85] Nishiya A.T., Massoco C.O., Felizzola C.R., Perlmann E., Batschinski K., Tedardi M.V., Garcia J.S., Mendonça P.P., Teixeira T.F., Dagli M.L.Z. (2016). Comparative Aspects of Canine Melanoma. Vet. Sci..

[86] Di Palma S., McConnell A., Verganti S., Starkey M. (2021). Review on Canine Oral Melanoma: An Undervalued Authentic Genetic Model of Human Oral Melanoma?. Vet. Pathol..

[87] Teixeira T.F., Gentile L.B., Da Silva T.C., Mennecier G., Chaible L.M., Cogliati B., Roman M.A.L., Gioso M.A., Dagli M.L.Z. (2014). Cell Proliferation and Expression of Connexins Differ in Melanotic and Amelanotic Canine Oral Melanomas. Vet. Res. Commun..

[88] Spangler W.L., Kass P.H. (2006). The Histologic and Epidemiologic Bases for Prognostic Considerations in Canine Melanocytic Neoplasia. Vet. Pathol..

[89] Shuman A.G., Light E., Olsen S.H., Pynnonen M.A., Taylor J.M.G., Johnson T.M., Bradford C.R. (2011). Mucosal Melanoma of the Head and Neck: Predictors of Prognosis. Arch. Otolaryngol. Head. Neck Surg..

[90] Millanta F., Fratini F., Corazza M., Castagnaro M., Zappulli V., Poli A. (2002). Proliferation Activity in Oral and Cutaneous Canine Melanocytic Tumours: Correlation with Histological Parameters, Location, and Clinical Behaviour. Res. Vet. Sci..

[91] Oh J.H., Cho J.Y. (2023). Comparative Oncology: Overcoming Human Cancer through Companion Animal Studies. Exp. Mol. Med..

[92] Ranieri G., Gadaleta C.D., Patruno R., Zizzo N., Daidone M.G., Hansson M.G., Paradiso A., Ribatti D. (2013). A Model of Study for Human Cancer: Spontaneous Occurring Tumors in Dogs. Biological Features and Translation for New Anticancer Therapies. Crit. Rev. Oncol. Hematol..

[93] Lindblad-Toh K., Wade C.M., Mikkelsen T.S., Karlsson E.K., Jaffe D.B., Kamal M., Clamp M., Chang J.L., Kulbokas E.J., Zody M.C. (2005). Genome Sequence, Comparative Analysis and Haplotype Structure of the Domestic Dog. Nature.

[94] Broniec A., Goto M., Matsuki H. (2009). A Peculiar Phase Transition of Plasmalogen Bilayer Membrane under High Pressure. Langmuir.

[95] Aramaki S., Tsuge S., Islam A., Eto F., Sakamoto T., Oyama S., Li W., Zhang C., Yamaguchi S., Takatsuka D. (2023). Lipidomics-Based Tissue Heterogeneity in Specimens of Luminal Breast Cancer Revealed by Clustering Analysis of Mass Spectrometry Imaging: A Preliminary Study. PLoS ONE.

[96] Wu Q., Yu J., Zhang M., Xiong Y., Zhu L., Wei B., Wu T., Du Y. (2024). Serum Lipidomic Profiling for Liver Cancer Screening Using Surface-Assisted Laser Desorption Ionization MS and Machine Learning. Talanta.

[97] Bifarin O.O., Sah S., Gaul D.A., Moore S.G., Chen R., Palaniappan M., Kim J., Matzuk M.M., Fernández F.M. (2023). Machine Learning Reveals Lipidome Remodeling Dynamics in a Mouse Model of Ovarian Cancer. J. Proteome Res..

[98] Xiao Y., Ma D., Yang Y.S., Yang F., Ding J.H., Gong Y., Jiang L., Ge L.P., Wu S.Y., Yu Q. (2022). Comprehensive Metabolomics Expands Precision Medicine for Triple-Negative Breast Cancer. Cell Res..

[99] Li R., Li L., Xu Y., Yang J. (2022). Machine Learning Meets Omics: Applications and Perspectives. Brief. Bioinform..

[100] Cai Z., Poulos R.C., Liu J., Zhong Q. (2022). IScience Machine Learning for Multi-Omics Data Integration in Cancer. iScience.

[101] Arjmand B., Hamidpour S.K., Tayanloo-Beik A., Goodarzi P., Aghayan H.R., Adibi H., Larijani B. (2022). Machine Learning: A New Prospect in Multi-Omics Data Analysis of Cancer. Front. Genet..

[102] Csala A., Zwinderman A.H. (2019). Multivariate Statistical Methods for High-Dimensional Multiset Omics Data Analysis. Comput. Biol..

[103] Caponigro V., Tornesello A.L., Merciai F., La Gioia D., Salviati E., Basilicata M.G., Musella S., Izzo F., Megna A.S., Buonaguro L. (2023). Integrated Plasma Metabolomics and Lipidomics Profiling Highlights Distinctive Signature of Hepatocellular Carcinoma in HCV Patients. J. Transl. Med..

[104] Wolrab D., Jirásko R., Cífková E., Höring M., Mei D., Chocholoušková M., Peterka O., Idkowiak J., Hrnčiarová T., Kuchař L. (2022). Lipidomic Profiling of Human Serum Enables Detection of Pancreatic Cancer. Nat. Commun..

[105] Jiang N., Zhang Z., Chen X., Zhang G., Wang Y., Pan L., Yan C., Yang G., Zhao L., Han J. (2021). Plasma Lipidomics Profiling Reveals Biomarkers for Papillary Thyroid Cancer Diagnosis. Front. Cell Dev. Biol..

[106] Kim H.Y., Lee H., Kim S.H., Jin H., Bae J., Choi H.K. (2017). Discovery of Potential Biomarkers in Human Melanoma Cells with Different Metastatic Potential by Metabolic and Lipidomic Profiling. Sci. Rep..

[107] Kurokawa G.A., Hamamoto Filho P.T., Delafiori J., Galvani A.F., de Oliveira A.N., Dias-Audibert F.L., Catharino R.R., Pardini M.I.M.C., Zanini M.A., Lima E. (2023). de O.; et al. Differential Plasma Metabolites between High- and Low-Grade Meningioma Cases. Int. J. Mol. Sci..

[108] Gallart-Ayala H., Courant F., Severe S., Antignac J.P., Morio F., Abadie J., Le Bizec B. (2013). Versatile Lipid Profiling by Liquid Chromatography-High Resolution Mass Spectrometry Using All Ion Fragmentation and Polarity Switching. Preliminary Application for Serum Samples Phenotyping Related to Canine Mammary Cancer. Anal. Chim. Acta.

[109] Safari Yazd H., Bazargani S.F., Fitzpatrick G., Yost R.A., Kresak J., Garrett T.J. (2023). Metabolomic and Lipidomic Characterization of Meningioma Grades Using LC-HRMS and Machine Learning. J. Am. Soc. Mass. Spectrom..

[110] Shang X., Zhang C., Kong R., Zhao C., Wang H. (2024). Construction of a Diagnostic Model for Small Cell Lung Cancer Combining Metabolomics and Integrated Machine Learning. Oncologist.

[111] Starodubtseva N.L., Tokareva A.O., Rodionov V.V., Brzhozovskiy A.G., Bugrova A.E., Chagovets V.V., Kometova V.V., Kukaev E.N., Soares N.C., Kovalev G.I. (2023). Integrating Proteomics and Lipidomics for Evaluating the Risk of Breast Cancer Progression: A Pilot Study. Biomedicines.

[112] Manzi M., Palazzo M., Knott M.E., Beauseroy P., Yankilevich P., Giménez M.I., Monge M.E. (2021). Coupled Mass-Spectrometry-Based Lipidomics Machine Learning Approach for Early Detection of Clear Cell Renal Cell Carcinoma. J. Proteome Res..

[113] Wang G., Yao H., Gong Y., Lu Z., Pang R., Li Y., Yuan Y., Song H., Liu J., Jin Y. (2021). Metabolic Detection and Systems Analyses of Pancreatic Ductal Adenocarcinoma through Machine Learning, Lipidomics, and Multi-Omics. Sci. Adv..

[114] Yang C., Zhou S., Zhu J., Sheng H., Mao W., Fu Z., Chen Z. (2022). Plasma Lipid-Based Machine Learning Models Provides a Potential Diagnostic Tool for Colorectal Cancer Patients. Clin. Chim. Acta.

[115] Krishnan S.T., Winkler D., Creek D., Anderson D., Kirana C., Maddern G.J., Fenix K., Hauben E., Rudd D., Voelcker N.H. (2023). Staging of Colorectal Cancer Using Lipid Biomarkers and Machine Learning. Metabolomics.

[116] Zhou J., Ji N., Wang G., Zhang Y., Song H., Yuan Y., Yang C., Jin Y., Zhang Z., Zhang L. (2022). Metabolic Detection of Malignant Brain Gliomas through Plasma Lipidomic Analysis and Support Vector Machine-Based Machine Learning. EBioMedicine.

[117] Saito R., Yoshimura K., Shoda K., Furuya S., Akaike H., Kawaguchi Y., Murata T., Ogata K., Iwano T., Takeda S. (2021). Diagnostic Significance of Plasma Lipid Markers and Machine Learning-Based Algorithm for Gastric Cancer. Oncol. Lett..

[118] Liu Z.C., Wu W.H., Huang S., Li Z.W., Li X., Shui G.H., Lam S.M., Li B.W., Li Z.X., Zhang Y. (2022). Plasma Lipids Signify the Progression of Precancerous Gastric Lesions to Gastric Cancer: A Prospective Targeted Lipidomics Study. Theranostics.

[119] O’Donnell V.B., Fitzgerald G.A., Murphy R.C., Liebisch G., Dennis E.A., Quehenberger O., Subramaniam S., Wakelam M.J.O. (2020). Steps Toward Minimal Reporting Standards for Lipidomics Mass Spectrometry in Biomedical Research Publications. Circ. Genom. Precis. Med..

[120] Garate J., Lage S., Martín-Saiz L., Perez-Valle A., Ochoa B., Boyano M.D., Fernández R., Fernández J.A. (2020). Influence of Lipid Fragmentation in the Data Analysis of Imaging Mass Spectrometry Experiments. J. Am. Soc. Mass. Spectrom..

[121] Wolrab D., Jirásko R., Peterka O., Idkowiak J., Chocholoušková M., Vaňková Z., Hořejší K., Brabcová I., Vrána D., Študentová H. (2021). Plasma Lipidomic Profiles of Kidney, Breast and Prostate Cancer Patients Differ from Healthy Controls. Sci. Rep..

[122] Holcapek M., Cifkova E., Lisa M., Jirasko R., Wolrab D., Hrnciarová T. (2018). A Method of Diagnosing Pancreatic Cancer Based on Lipidomic Analysis of a Body Fluid.

[123] Garate J., Lage S., Fernández R., Velasco V., Abad B., Asumendi A., Gardeazabal J., Arroyo-Berdugo Y., Rodríguez M.Á., Artola J.L. (2019). Imaging Mass Spectrometry-Based Lipidomic Approach to Classification of Architectural Features in Nevi. J. Invest. Dermatol..

[124] Asumendi Mallea A., Boyano López M.D., Barreda Gómez G., Astigarraga Arribas E., Fernández González J.A., Ochoa Olascoaga M.B. (2022). Method for the Diagnosis of Melanoma.

[125] Kujala M., Nevalainen J. (2015). A Case Study of Normalization, Missing Data and Variable Selection Methods in Lipidomics. Stat. Med..

[126] Del Prete E., Campos A.M., Della Rocca F., Gallo C., Fontana A., Nuzzo G., Angelini C. (2022). ADViSELipidomics: A Workflow for Analyzing Lipidomics Data. Bioinformatics.

[127] Ding X., Yang F., Chen Y., Xu J., He J., Zhang R., Abliz Z. (2022). Norm ISWSVR: A Data Integration and Normalization Approach for Large-Scale Metabolomics. Anal. Chem..

[128] Ulmer C.Z., Ragland J.M., Koelmel J.P., Heckert A., Jones C.M., Garrett T.J., Yost R.A., Bowden J.A. (2017). LipidQC: Method Validation Tool for Visual Comparison to SRM 1950 Using NIST Interlaboratory Comparison Exercise Lipid Consensus Mean Estimate Values. Anal. Chem..

[129] Köfeler H.C., Ahrends R., Baker E.S., Ekroos K., Han X., Hoffmann N., Holcapek M., Wenk M.R., Liebisch G. (2021). Recommendations for Good Practice in Ms-Based Lipidomics. J. Lipid Res..

[130] Triebl A., Burla B., Selvalatchmanan J., Oh J., Tan S.H., Chan M.Y., Mellet N.A., Meikle P.J., Torta F., Wenk M.R. (2020). Shared Reference Materials Harmonize Lipidomics across MS-Based Detection Platforms and Laboratories. J. Lipid Res..

[131] Tsuchida S., Nakayama T. (2022). MALDI-Based Mass Spectrometry in Clinical Testing: Focus on Bacterial Identification. Appl. Sci..

[132] Poirion O.B., Jing Z., Chaudhary K., Huang S., Garmire L.X. (2021). DeepProg: An Ensemble of Deep-Learning and Machine-Learning Models for Prognosis Prediction Using Multi-Omics Data. Genome Med..

[133] Huang L., Song M., Shen H., Hong H., Gong P., Deng H.-W., Zhang C. (2023). Deep Learning Methods for Omics Data Imputation. Biology.

[134] Kang M., Ko E., Mersha T.B. (2022). A Roadmap for Multi-Omics Data Integration Using Deep Learning. Brief. Bioinform..

[135] Albaradei S., Thafar M., Alsaedi A., Van Neste C., Gojobori T., Essack M., Gao X. (2021). Machine Learning and Deep Learning Methods That Use Omics Data for Metastasis Prediction. Comput. Struct. Biotechnol. J..

[136] Tran K.A., Kondrashova O., Bradley A., Williams E.D., Pearson J.V., Waddell N. (2021). Deep Learning in Cancer Diagnosis, Prognosis and Treatment Selection. Genome Med..

[137] Babu M., Snyder M. (2023). Multi-Omics Profiling for Health. Mol. Cell Proteom..

[138] Gómez-Cebrián N., Poveda J.L., Pineda-Lucena A., Puchades-Carrasco L. (2022). Metabolic Phenotyping in Prostate Cancer Using Multi-Omics Approaches. Cancers.

[139] Alvarez-Frutos L., Barriuso D., Duran M., Infante M., Kroemer G., Palacios-Ramirez R., Senovilla L. (2023). Multiomics Insights on the Onset, Progression, and Metastatic Evolution of Breast Cancer. Front. Oncol..

[140] Kiebish M.A., Cullen J., Mishra P., Ali A., Milliman E., Rodrigues L.O., Chen E.Y., Tolstikov V., Zhang L., Panagopoulos K. (2020). Multi-Omic Serum Biomarkers for Prognosis of Disease Progression in Prostate Cancer. J. Transl. Med..

[141] Lima A.R., Carvalho M., Aveiro S.S., Melo T., Domingues M.R., Macedo-Silva C., Coimbra N., Jerónimo C., Henrique R., Bastos M.d.L. (2022). Comprehensive Metabolomics and Lipidomics Profiling of Prostate Cancer Tissue Reveals Metabolic Dysregulations Associated with Disease Development. J. Proteome Res..

[142] Jung J.H., Yang D.Q., Song H., Wang X., Wu X., Kim K.P., Pandey A., Byeon S.K. (2023). Characterization of Lipid Alterations by Oncogenic PIK3CA Mutations Using Untargeted Lipidomics in Breast Cancer. OMICS.

